# A novel small-angle neutron scattering detector geometry

**DOI:** 10.1107/S0021889813011862

**Published:** 2013-07-04

**Authors:** Kalliopi Kanaki, Andrew Jackson, Richard Hall-Wilton, Francesco Piscitelli, Oliver Kirstein, Ken H. Andersen

**Affiliations:** a European Spallation Source ESS AB, PO Box 176, 22 100 Lund, Sweden; b Institut Laue-Langevin, Grenoble, France

**Keywords:** boron-10, boron carbide, detectors, European Spallation Source (ESS), helium-3, geometry, neutrons, optimization based on material properties, small-angle neutron scattering (SANS)

## Abstract

A novel 2π detector geometry for small-angle neutron scattering (SANS) applications is presented and its theoretical performance evaluated. Such a novel geometry is ideally suited for a SANS instrument at the European Spallation Source (ESS). Motivated by the low availability and high price of ^3^He, the new concept utilizes gaseous detectors with ^10^B as the neutron converter. The shape of the detector is inspired by an optimization process based on the properties of the conversion material. Advantages over the detector geometry traditionally used on SANS instruments are discussed. The angular and time resolutions of the proposed detector concept are shown to satisfy the requirements of the particular SANS instrument.

## Abstract 2

A novel 2π detector geometry for small-angle neutron scattering (SANS) applications is presented and its theoretical performance evaluated. The shape of the detector is inspired by an optimization process based on the properties of the conversion material. Advantages over the detector geometry traditionally used on SANS instruments are discussed.

## 1. Introduction

The European Spallation Source (ESS) in Lund, Sweden, aspires to becoming the world’s leading neutron source with unprecedented intensity and brightness (ESS official web site, http://www.europeanspallationsource.se; Peggs *et al.*, 2012 ▶). By the end of its construction in 2025 ▶ a suite of 22 neutron scattering instruments will be available to the user community, with seven of them coming online in 2019 ▶. The ESS neutron pulse is going to be 2.86 ms long with a repetition rate of 14 Hz (Peggs *et al.*, 2012 ▶, 2013 ▶). A reference suite of neutron scattering instruments is currently being evaluated and it is envisaged that at least two SANS instruments will be constructed.

SANS is a technique to obtain structural information with a broad range of applicability across soft matter, biology, condensed matter and materials science research, which makes SANS instruments some of the most common and highly demanded instruments found at neutron sources around the world [D22 at ILL (http://www.ill.eu/instruments-support/instruments-groups/instruments/d22/description/instrument-layout/), KWS at FRM-II (http://www.frm2.tum.de/wissenschaftliche-nutzung/diffraktion/kws-1/index.html), Sans2d at ISIS (http://www.isis.stfc.ac.uk/instruments/sans2d/sans2d3000.html)]. This paper focuses on how the particular instrument characteristics determine the technology choices and optimization process for detector design. The relevant requirements for one of the ESS SANS instruments currently being investigated are a broad *Q* range, where 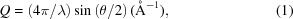
(λ is the neutron wavelength and θ is the scattering angle), simultaneously accessible in a single measurement (10^−3^ < *Q* < 1 Å^−1^) and a detector polar-angle resolution δθ/θ of 10% or better. In the following sections a novel detector concept is described that fulfils the aforementioned conditions, whilst vastly improving the data-collection efficiency over present SANS instruments. The main technical characteristics of such a detector are calculated analytically and discussed.

## 2. The novel detector geometry

**Figure 1:**
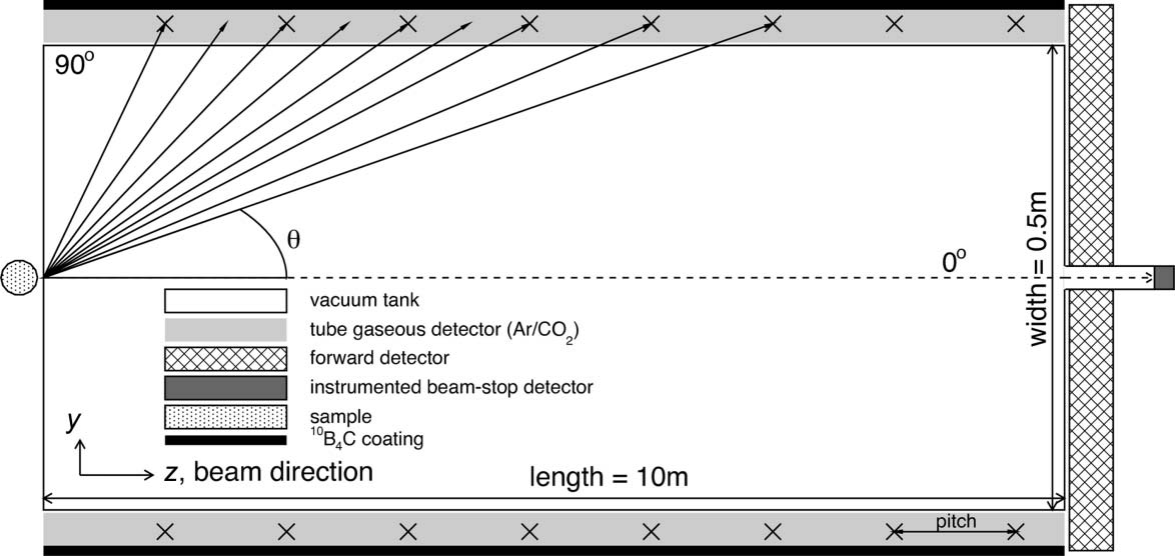
Cross section of the proposed detector geometry on the *yz* plane (dimensions not to scale). The attainable polar angle θ ranges from 0 to 90°, with the lowest possible angle of the tube being 1.4° for the chosen detector dimensions. The lines connecting the sample and the anode wires (crosses) represent possible scattered neutron trajectories. The line from the centre of the tube to the centre of the wire defines the azimuthal angle.

Typically, SANS instruments at neutron sources such as reactors or spallation sources have used a single position-sensitive moveable detector to enable a wide *Q* range to be examined. There is now a trend towards using multiple detector banks to access a greater range of scattering angle at the same time [NOMAD at SNS (http://neutrons.ornl.gov/nomad/), Nimrod (http://www.isis.stfc.ac.uk/instruments/nimrod/) and SANDALS (http://www.isis.stfc.ac.uk/instruments/sandals) at ISIS], thus facilitating measurement and reducing the data-collection time. The alternative detector concept proposed here extrapolates this trend to its logical conclusion. It consists of a tube-like geometry with a polyhedral cross section (see Figs. 1 ▶ and 2 ▶), providing a 2π solid angle coverage by utilizing two segmented detectors, a 10 m-long and 0.5 m-wide tube detector and a forward detector. The attention of this paper is focused on the tube segment, since the forward detector resembles the traditional SANS detector approach.

The advantage of the tube-like geometry is immediately apparent, as a large solid angle and consequently a wide *Q* range are readily accessible (see Fig. 3 ▶) without the need to interrupt the measurement and shift the detectors up- or downstream. The detectors and their readout electronics can be installed outside the vacuum tank, leading to a smaller physical size for the latter, which reduces not only the technical challenges but also, potentially, the cost of the setup. Furthermore, the possibility of filling the vacuum tank with a noble gas could be explored, since a gas pressure closer to that of the detector gas eliminates the need for a thick tank wall or even advocates its replacement with a thin foil separating the two gas volumes.

More importantly though, the choice of the detector geometry exploits the material properties of the neutron converter. Since the traditional approach with ^3^He detectors is no longer a viable option for ESS instruments, owing to the unavailability of ^3^He, particularly for the ESS, and its high cost (Persons & Aloise, 2011 ▶; Shea & Morgan, 2010 ▶; Cho, 2009 ▶; International Collaboration on the Development of Neutron Detectors, http://www.icnd.org), an alternative neutron technology solution based on ^10^B is introduced for this application. Boron carbide (^10^B_4_C) thin-film detectors are currently being developed by several groups worldwide and are proven to be a reliable and promising replacement for ^3^He detectors (Andersen *et al.*, 2012 ▶; Zeitelhack, 2012 ▶; Bigault *et al.*, 2012 ▶; Correa, 2012 ▶; Stefanescu *et al.*, 2013 ▶; Höglund *et al.*, 2012 ▶). The ^10^B isotope has a neutron absorption cross section of about 3840 barn (1 barn = 10^−28^ m^2^) for thermal neutrons (λ = 1.8 Å) (Safford *et al.*, 1960 ▶), which is approximately 70% of the ^3^He cross section. Boron itself is an insulator, but in the form of deposited ^10^B_4_C it becomes a conductor and therefore an appropriate choice for a cathode material.

In the description assumed here, a thin layer of ^10^B_4_C, enriched in ^10^B to more than 95% (Höglund *et al.*, 2012 ▶), coats the inner aluminium walls of the active detector area with a typical coating thickness of 1 µm. The scattered neutrons traversing the thin film convert with a certain probability, depending on their wavelength. The products of the capture process, an α particle and a ^7^Li ion, are emitted in opposite directions, 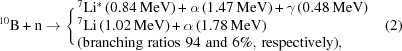
with energies large enough to allow micrometre-long ranges in the ^10^B_4_C film and eventually large enough to escape from the layer, provided the thickness of the latter is optimized for this purpose. As a result of the back-to-back emission, only one of the products has the possibility of entering the gas detection area. The other particle is stopped, either inside the converter layer or inside the aluminium substrate on which the converter is coated. Provided one of the charged products exits the layer with sufficient energy left, it ionizes the detector gas, and the signal produced is amplified by an electric field and finally collected by anode wires, as in a typical multi-wire proportional chamber (MWPC) (Charpak *et al.*, 1968 ▶; Charpak & Sauli, 1984 ▶).

It becomes apparent that the detector design is a multi-parameter optimization problem, the parameters being, for example, the dimensions and total surface of the detector, the thickness of the ^10^B_4_C layer, the anode wire pitch, the gas composition, and the gain, to mention just a few. The motivation for this work is to present a feasibility study of a design concept, rather than a detailed and mature design. In that spirit, analytical calculations are used as a first step to this end, aimed at demonstrating whether this detector concept suits the instrument needs and how it can be further optimized.

## 3. Evaluation of the tube detector efficiency

The elongated shape of the tube detector causes the neutrons to intersect the conversion layer at shallow incident angles (<10°) at a distance of only 1–2 m from the sample. Small incident angles lead to longer neutron paths through the ^10^B_4_C layer and to a higher conversion probability from algebraic considerations. Moreover, with a suitable optimization of the layer thickness, the escape probability of the ions can be increased as well. The combination of the two effects results in a high detection efficiency. The idea of enhancing detection efficiency by reducing the neutron incident angle has been used successfully by other groups as well (Henske *et al.*, 2012 ▶; Piscitelli *et al.*, 2012 ▶; Nowak *et al.*, 2012 ▶).

The detection mode for the conversion products is the ‘back-scattering’ one (see Fig. 4 ▶), since it results in a fundamentally better efficiency than the transmission mode (see Fig. 5 ▶). For cold-neutron detection in particular, the back-scattering mode is the most suitable option for obtaining a high detection efficiency.

Since the neutrons always hit the converter layer at small angles over almost the entire surface, and at such angles the measurements are of higher relevance for the SANS method, the efficiency values are well above 50% in the areas where the detector occupancy, *i.e.* the fraction of hits per channel or unit area, is high. The largest part of the difference between the detection of an Li ion and an α particle is accounted for in the algebraic efficiency calculations, as the two particles have different ranges in the coating layer. For the purposes of this study, the intrinsic detection efficiency of the gaseous detector is reasonably assumed to be very high (Sauli, 1977 ▶; Klein, 2000 ▶; Stefanescu *et al.*, 2013 ▶), with every ion escape leading to a detection event. The conversion layer thickness can be optimized to increase the back-scattering detection efficiency further, as shown in Fig. 6 ▶ (Piscitelli & van Esch, 2013 ▶), but the optimization procedure for this parameter is not addressed in this paper.

## 4. Real space and time resolution of the tube detector

Apart from the high detection efficiency, the elongated geometry of the tube detector results in an excellent polar angle (θ) resolution. The shallow angle of the incident neutrons throughout the largest part of the detector is responsible for the very small polar-angle differences between neutrons whose conversion products are detected between consecutive wires (see Fig. 1 ▶); the result is a significant resolution improvement compared with what can be achieved with the traditional geometry, where the incident neutron hits the detector perpendicularly.

In addition to that, the traditional parallax effects of a ^3^He tube are almost completely eliminated with the use of a boron-coated MWPC. For a 5 cm-thick ^3^He tube and a neutron incident angle of 10°, the total possible path of the neutron is about 28 cm. In the case of the tube detector, the same incident angle results in a 5.7 µm path for a 1 µm converter thickness. Thus, the tube detector overcomes the parallax effect, as the possible absorption points are far more localized in a thin ^10^B_4_C layer than in a large active gas volume. Any smearing is related to the range of any ions produced inside the MWPC gas, which is of the order of a few millimetres (Correa, 2012 ▶), although this is small compared with the wire pitches considered here.

Ignoring for the moment possible scattering of the neutrons at the walls of the vacuum tank, and assuming that the conversion products are detected by the wire nearest to the conversion point, the polar angle resolution on the *yz* plane may be expressed by the difference 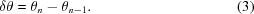
For this discussion the impact of the instrument collimator on the polar-angle resolution is not considered. The position resolution of the detector along the *z* axis is half the anode-wire pitch. The index *n* counts the half-pitch steps along the imaginary line that connects the wires, starting from the higher θ angles (90° perpendicular to the beam axis) and moving towards 0° (parallel to the beam axis). The impact of the wire pitch on the θ resolution is depicted in Fig. 7 ▶ for a 10 m-long and 0.5 m-wide detector. Already, very close to the entrance window of the detector near the sample, the δθ values are small enough to give δθ/θ of less than 10%, in agreement with the requirements discussed in §2. Furthermore, the wider wire pitch of 5 cm seems to be a suitable starting choice, bearing in mind that a lower number of readout channels is beneficial for the overall cost of the setup.

**Figure 2:**
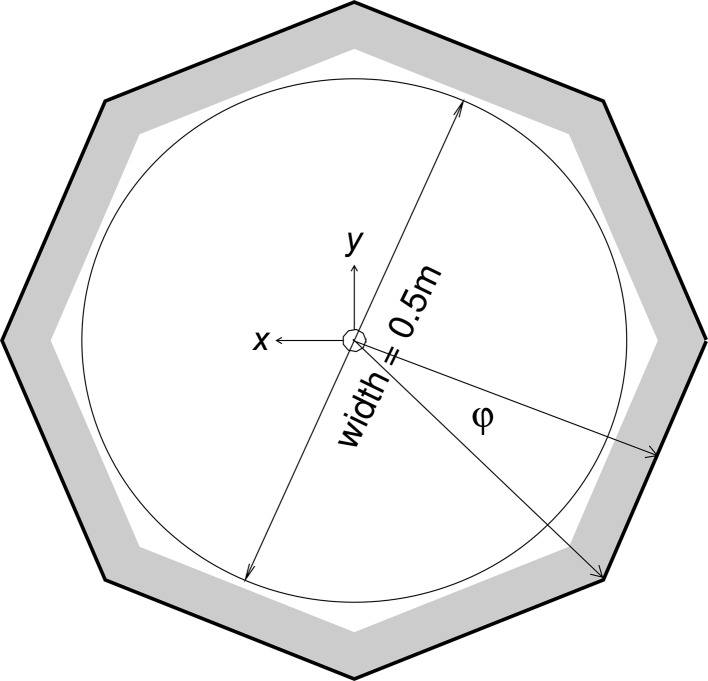
Cross section of the proposed detector geometry on the *xy* plane (dimensions not to scale).

The aforementioned calculations are performed without taking the azimuthal angle ϕ into consideration (see Fig. 2 ▶ for the definition of ϕ). The influence of ϕ on the θ resolution is depicted in Fig. 8 ▶ for a wire pitch of 5 cm. The δθ values for ϕ = 30° are compared with δθ for ϕ = 0°. It is clear that the impact of the ϕ angle on the polar-angle resolution is negligible, regardless of the number of faces of the detector cross section.

The ϕ resolution is determined by the number of faces of the tube detector. For an octagonal cross section it is 45° and for an hexagonal one it is 60°. This is acceptable for azimuthally symmetric scattering that can be averaged to a one-dimensional scattering curve, but is not suitable for studies of oriented systems where good azimuthal resolution is required. For such cases, the addition of a second set of perpendicular anode wires or a strip-type cathode readout could be considered, but the investigation of such a concept lies outside the scope of this paper.

Another aspect of the detector concept that can be calculated analytically is the time resolution based on the time-of-flight (TOF) differences between consecutive *n* points. Following the same definition as for the θ resolution, the TOF resolution along the *z* axis may be expressed as 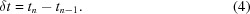
As expected, the arrival time differences are larger for a larger wire pitch and the separation between wavelengths for the same pitch increases with increasing pitch (see Fig. 9 ▶). The impact of the azimuthal angle on the time resolution is calculated to be negligible. The TOF resolution values of the tube detector are well below the timing uncertainties stemming from the ESS pulse length of 2.86 ms, so they do not pose a concern for the detector design *per se*.

## 5. *Q* range and resolution

**Figure 3:**
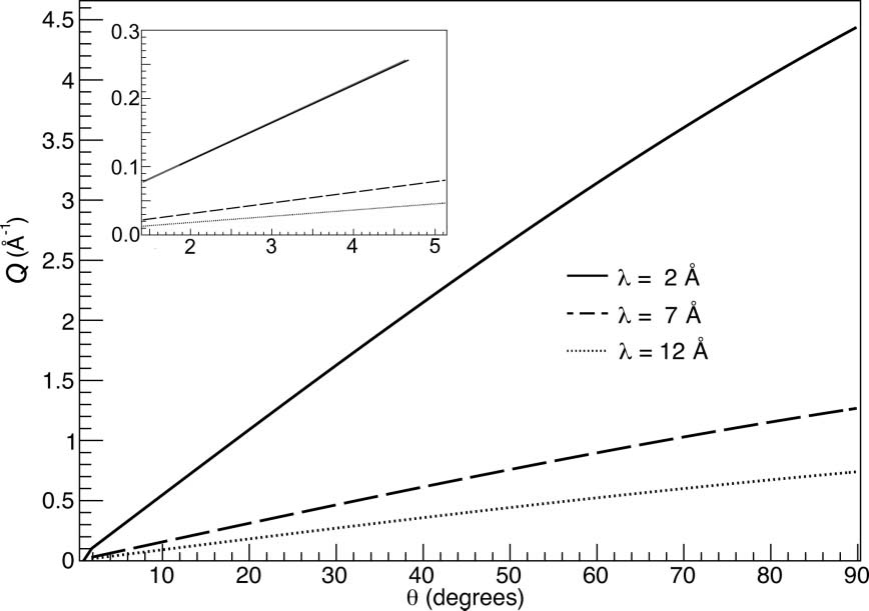
*Q* space available for the neutron wavelengths relevant to the SANS technique as a function of the polar angle, calculated for a 10 m-long and 0.5 m-wide tube. The lower accessible angle for these tube dimensions is 1.4°. Taking the forward detector into account, both the polar angle and *Q* approach zero.

**Figure 4:**
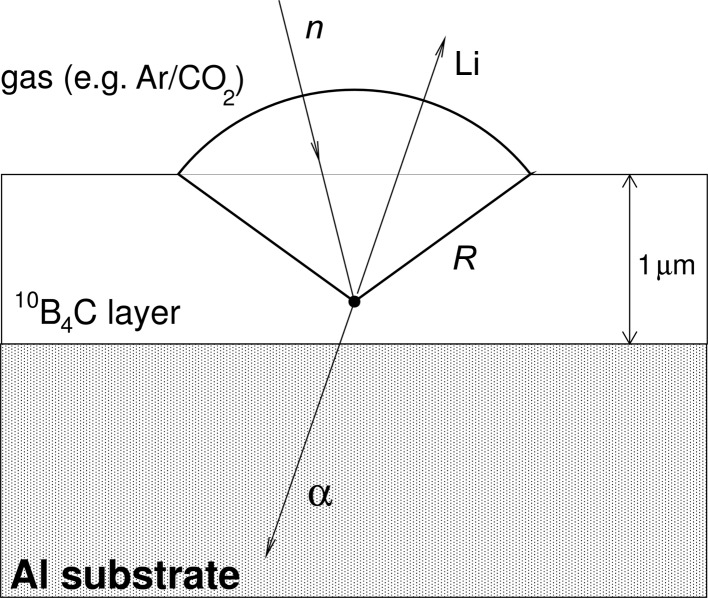
‘Back-scattering’ mode for neutron detection. The ionized conversion products are detected opposite to the direction of the incoming neutrons.

**Figure 5:**
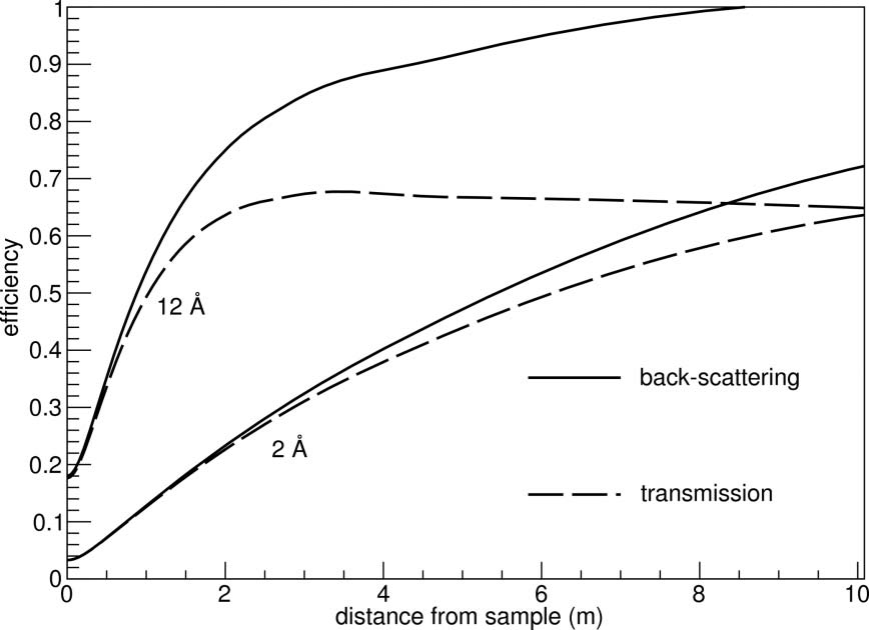
Detection efficiency of a single 1 µm-thick ^10^B_4_C layer, as a function of the distance from the sample on the *z* axis. Faster neutrons with lower wavelengths have a lower absorption cross section, hence the lower detection efficiency.

**Figure 6:**
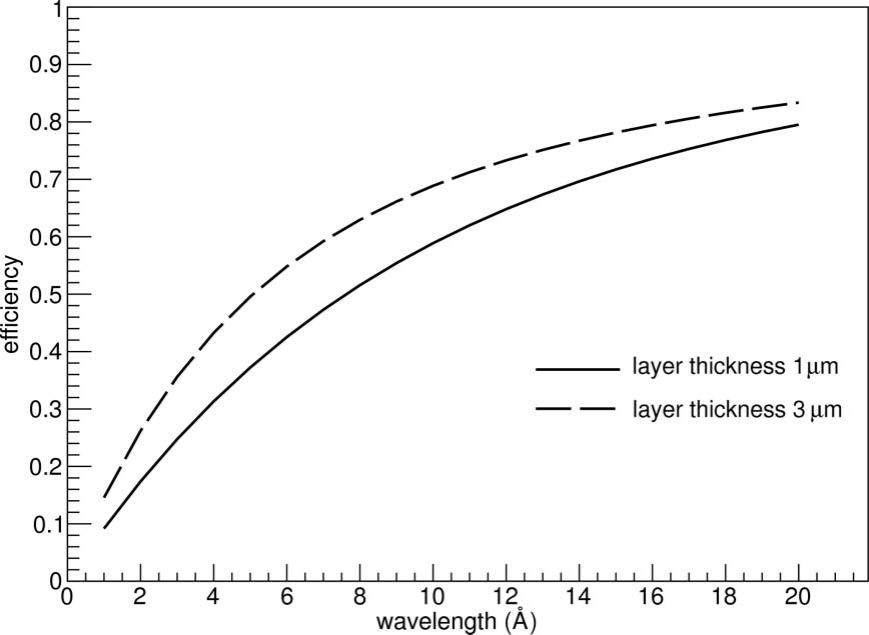
Detection back-scattering efficiency of a single ^10^B_4_C layer, as a function of neutron wavelength, for two layer thicknesses and an incident angle of 10°.

**Figure 7:**
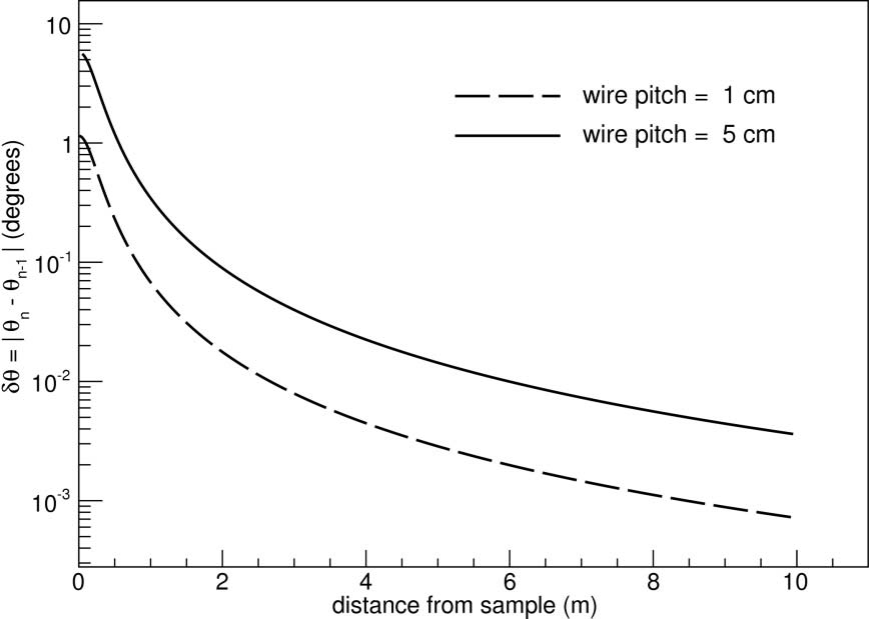
Polar angle resolution for 0° azimuthal angle, as a function of the distance from the sample on the *z* axis, for a 10 m-long and 0.5 m-wide detector and two wire pitch values.

**Figure 8:**
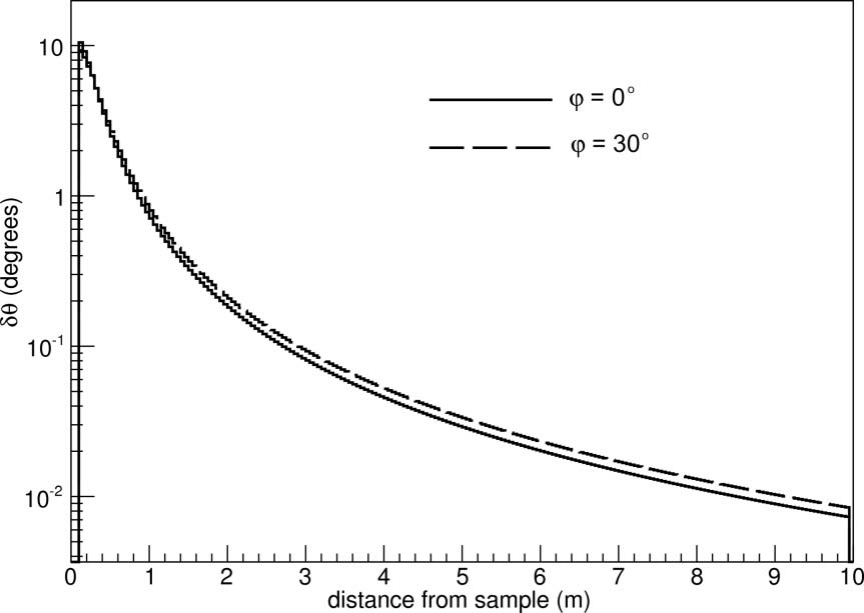
Impact of the ϕ angle on the θ resolution for a 10 m-long and 0.5 m-wide detector and a wire pitch of 5 cm. The range of the ϕ angle up to 30° covers both the octagonal and hexagonal cross-section scenarios.

**Figure 9:**
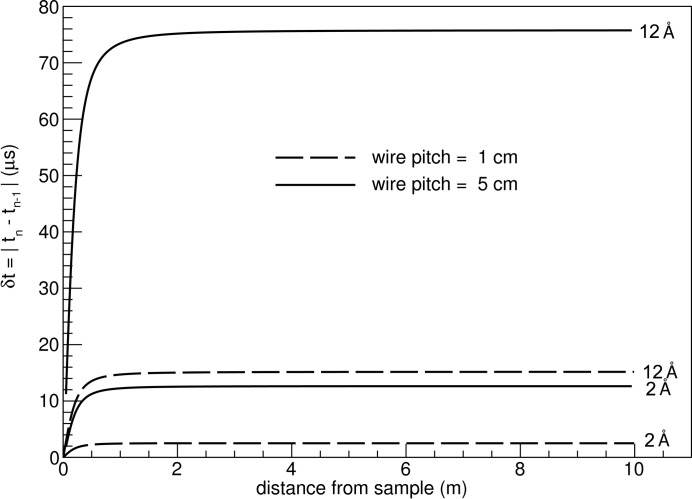
TOF resolution for a 10 m-long and 0.5 m-wide detector and wire pitches of 1 cm and 5 cm (ϕ = 0°).

Having calculated the real space and time characteristics of the tube detector, the next step is to translate these to the physics variables relevant to neutron scattering techniques. The momentum transfer *Q* relates to the scattering angle θ and the neutron wavelength λ according to equation (1). For the wavelengths used in SANS and the scattering angles accessible with this detector, the *Q* space accessible is depicted in Fig. 3 ▶ for a selection of wavelengths. The *Q* range satisfies the requirements of a typical instrument in this respect, thanks to the selected geometry.

**Figure 10:**
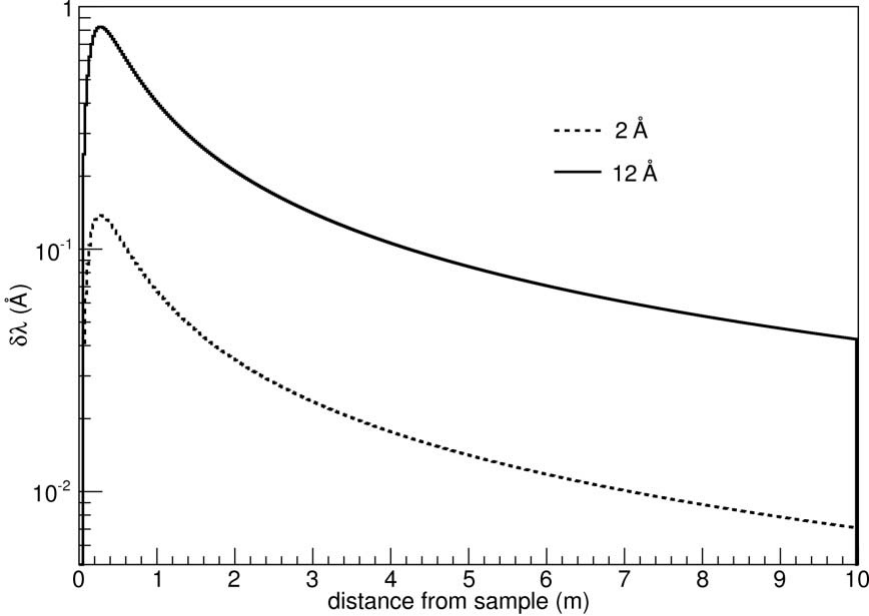
λ resolution in absolute values for 2 and 12 Å neutrons, as a function of distance from the sample.

The uncertainties in the determination of the polar angle and the neutron wavelength have an impact on the *Q* resolution. The latter is expressed as 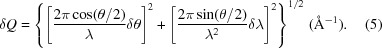
From the relation of λ to the time of flight *t*, and the distance *L* between the sample and the hit on the wire plane, the δλ resolution is calculated to be 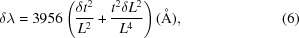
assuming that measured time and distance are independent variables. For the sake of the present discussion, the time of flight has been calculated using the distance of the neutron from the wire and its velocity. Propagating the resolution values for the polar angle, time of flight and distance, the λ and *Q* resolution dependencies as a function of the distance from the sample for ϕ = 0° are presented in Figs. 10 ▶–13 ▶ ▶ ▶. The influence of ϕ on these calculations is negligible, as mentioned earlier.

The calculation of *Q* resolution presented in equation (5) is the convolution of the detector angle resolution with the wavelength resolution due to the time of flight uncertainties, without taking into account the contributions of other instrument, beam and sample parameters. It is the theoretical intrinsic *Q* resolution of the tube detector. The improvement in angle resolution with the use of a tilted geometry translates to an improved *Q* resolution, as expressed in the first term of equation (5). Assuming the same spatial resolution of 1 cm for both the forward and tube detectors, the resulting polar-angle resolution is about 40 times higher for the tilted geometry at the point where the two detectors meet.

## 6. Background considerations

**Figure 14:**
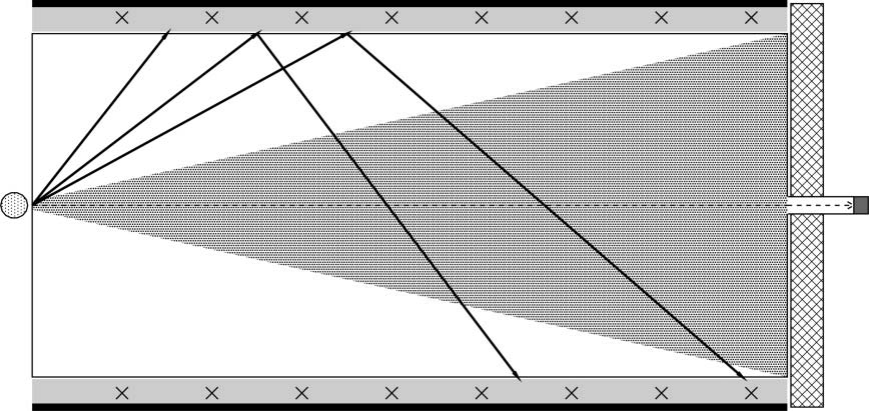
Conical structure for background reduction. Neutrons scattered on the vacuum tank walls are prevented from hitting the opposite side of the detector.

The proposed geometry might be prone to background arising from neutrons being scattered on one side of the detector and hitting an opposite or neighbouring side, giving rise to a mislocated signal, as illustrated in Fig. 14 ▶. Solutions to this problem exist. As an example, Fig. 14 ▶ depicts an absorbing conical structure that separates the vacuum tank into two parts. The exterior of the cone could contain a noble gas at normal atmospheric pressure, thus reducing the volume where the vacuum has to be maintained. Neutrons that travel towards the forward detector continue their course uninterrupted, while those scattered on the side walls of the vacuum tank are absorbed and prevented from creating a fake signal.

**Figure 15:**
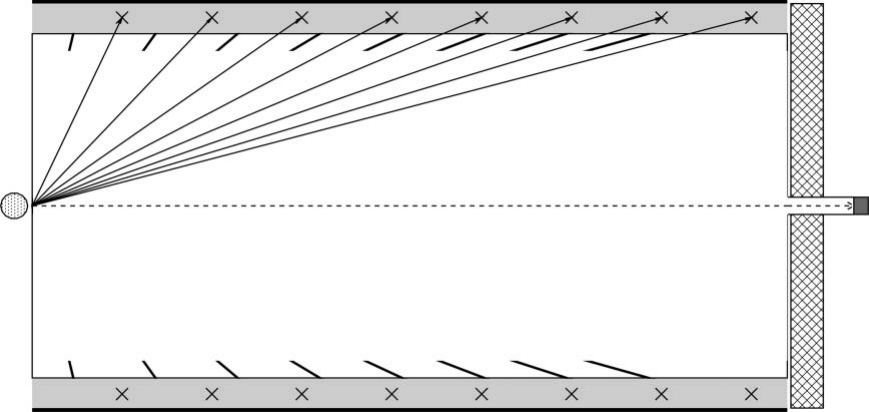
Geometric absorbers installed on the inner sides of the vacuum tank walls lower the number of scattered neutrons that hit the other side of the detector and add to the structural rigidity of the construction, thus allowing for thinner tank walls and further reduction of the scattering effects.

Another solution is proposed in Fig. 15 ▶. Geometric winglet-shaped absorbers, which might be thought of as flattened out radial collimators, are installed along the walls of the vacuum tank, covering full azimuthal angle. They act similarly to the cone structure, disallowing any direct view between a scattering point on the tank wall and the detection surface. Furthermore, they enhance the structural rigidity of the tank, leading to thinner walls and a subsequent reduction in scattering.

Both geometric arrangements reject a large proportion of the scattered neutrons. The latter one offers a better handle on the problem owing to better solid-angle shadowing. This will ensure that the background problem is not greater than with present SANS arrangements. Furthermore, in a realistic scenario two layers of detection could be used, exploiting the neutron energy-discrimination idea indicated by Hall-Wilton (2012 ▶). Detailed simulations and engineering design will provide a better description of the background and help qualify the most suitable solution for a particular implementation.

## 7. Conclusions

A new detector concept for a broadband SANS instrument was presented and its main characteristics evaluated by means of analytical calculations. A novel tube-like geometry, combining an alternative neutron converter and the principle of an MWPC, was discussed. The most important aspects of the detector performance were studied, with the aim of demonstrating the suitability of the concept for the particular application. The outcome of this study shows that the calculated resolutions, even with a preliminary choice of detector dimensions and wire pitch, fully satisfy the requirements of the proposed instrument from the detector point of view. The properties of the detector design, in combination with the apparent simplicity of the detection geometry and the potentially low cost, make it an appropriate choice for instruments at ESS and elsewhere.

## Figures and Tables

**Figure 11:**
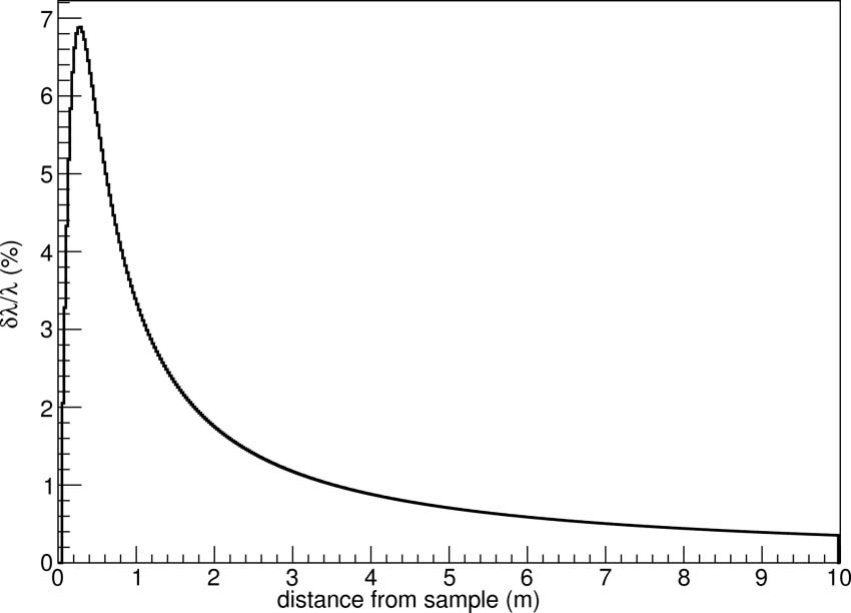
Normalized λ resolution as a function of distance from the sample. The wavelength dependence is cancelled out.

**Figure 12:**
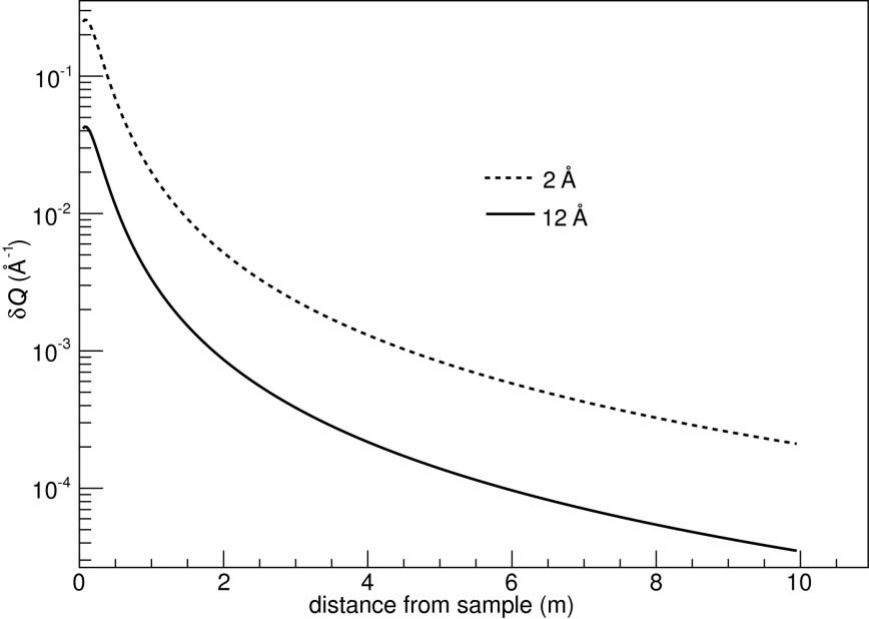
*Q* resolution for a 10 m-long and 0.5 m-wide tube detector, as a function of distance from the sample.

**Figure 13:**
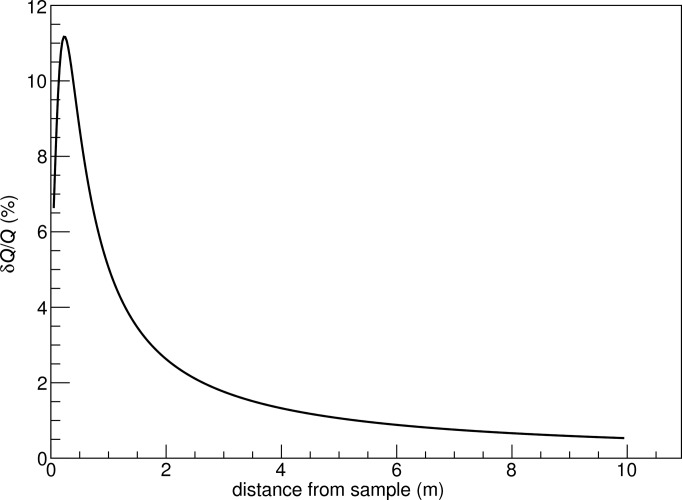
Normalized *Q* resolution for a 10 m-long and 0.5 m-wide tube detector, as a function of distance from the sample.
