# A novel small-angle neutron scattering detector geometry. Corrigendum

**DOI:** 10.1107/S0021889813022826

**Published:** 2013-09-11

**Authors:** Kalliopi Kanaki, Andrew Jackson, Richard Hall-Wilton, Francesco Piscitelli, Oliver Kirstein, Ken H. Andersen

**Affiliations:** a European Spallation Source ESS AB, PO Box 176, 22 100 Lund, Sweden; b Institut Laue–Langevin, Grenoble, France

**Keywords:** boron-10, boron carbide, detectors, European Spallation Source (ESS), helium-3, geometry, neutrons, optimization based on material properties, small-angle neutron scattering (SANS)

## Abstract

Errors in the paper by Kanaki, Jackson, Hall-Wilton, Piscitelli, Kirstein & Andersen [*J. Appl. Cryst.* (2013), **46**, 1031–1037] are corrected.

In equation (5) of Kanaki *et al.* (2013 ▶), a factor of 2 is missing from the wavelength contribution to the *Q* resolution. The correct equation is
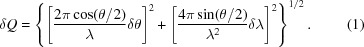



Figs. 12 and 13 of Kanaki *et al.* (2013 ▶) are updated as Figs. 1 ▶ and 2 ▶ of the current article to reflect the correction.

In equation (6) of Kanaki *et al.* (2013 ▶), the brackets are missing a square root. The correct version is
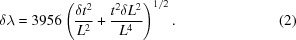



## Figures and Tables

**Figure 1:**
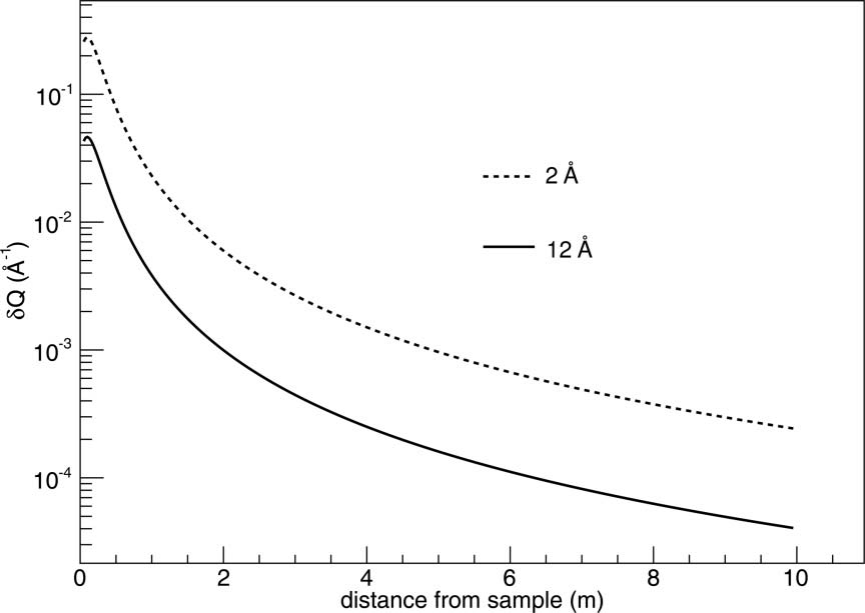
*Q* resolution for a 10 m-long and 0.5 m-wide tube detector, as a function of distance from the sample.

**Figure 2:**
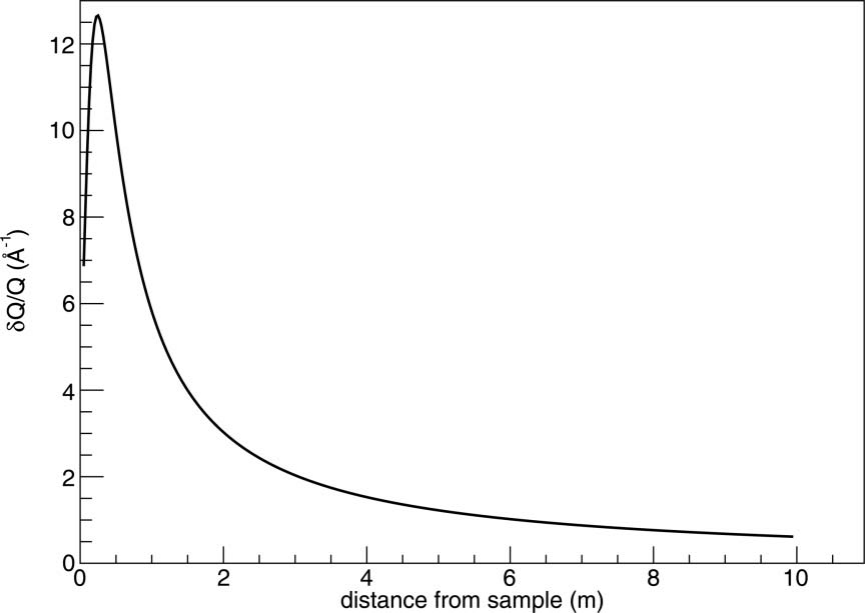
Normalized *Q* resolution for a 10 m-long and 0.5 m-wide tube detector, as a function of distance from the sample.
